# En Bloc Surgical Dissection for Penile Transplantation for Trans-Men: A Cadaveric Study

**DOI:** 10.1155/2018/6754030

**Published:** 2018-05-08

**Authors:** Gennaro Selvaggi, Erica Wesslen, Anna Elander, Peter Wroblewski, Andri Thorarinsson, Michael Olausson

**Affiliations:** ^1^Department of Plastic Surgery, Institute of Clinical Sciences, Sahlgrenska Academy, University of Gothenburg, Sahlgrenska University Hospital, Göteborg, Sweden; ^2^Department of Transplantation Surgery, Institute of Clinical Sciences, Sahlgrenska Academy, University of Gothenburg, Sahlgrenska University Hospital, Göteborg, Sweden

## Abstract

**Introduction:**

The surgical techniques currently available for penile reconstruction for trans-men with gender dysphoria present with multiple drawbacks and often fail to meet patients' expectations. Literature reports three cases where penile transplantation has been performed for cis-men, with the last two cases being considered successful.

**Aim:**

To determine whether an en bloc surgical dissection can be performed in a male cadaver, in order to include structures necessary for penile transplantation (from a deceased donor male) to a recipient with female genitalia in gender affirmation surgery.

**Method:**

The study was conducted in the form of explorative dissections of the genital and pelvic regions of three male cadavers preserved in phenol-ethanol solution.

**Results:**

The first two dissections failed to explant adequately all the relevant structures. The third dissection, which was performed along the pubic arch and through the perineum, succeeded in explanting the relevant structures: it, in fact, allowed for identification and adequate transection of urethra, vessels, dorsal nerves, crura of corpora cavernosa, and bulb of corpus spongiosum, in en bloc explantation of male genitalia.

**Conclusions:**

It is possible to explant the penis and associated vessels, nerves, and urethra en bloc from a cadaver. This study suggests a surgical technique for en bloc explantation aiming for transplantation of the penis from a cadaveric donor male to a recipient with female genitalia.

## 1. Introduction

In gender affirmation surgery (GAS) trans-men patients chose which surgical technique or penis reconstruction best corresponds to their wishes. The choice is normally taken in accordance and following discussion with the surgeon and possibly the mental health professionals [1, 2]. However, for some of the patients the surgical techniques currently available (e.g., phalloplasty with radial forearm flap, thigh flap, and metoidioplasty) are not adequate. In addition, some patients will incur in complications that will result in poorly functioning genitalia, or simply the outcomes will not be satisfactory [3, 4]. All the options available today for penile reconstruction for trans-men, in fact, present with multiple drawbacks, with a relative lack of scientific data regarding complications and results [5, 6]; in addition, dissatisfaction and regret after SRS, though infrequent, are correlated with a poor surgical outcome [7]. Thus, there is a need to explore new surgical options for penile reconstruction for trans-men. Already in 2006, it was argued that transplantation could have advantages compared to the current available techniques [8]; if successful, in fact, a penile transplant has a chance of providing a cosmetic and functional result superior to that of a surgically constructed neophallus.

Recent literature reports three allogenic human penile transplantations [9] performed in cis-men: therefore, none of them was performed for GAS. The first one was performed in China in 2006 on a man after traumatic severance of the penis, with the surgery reversed after two weeks due to a negative psychological reaction [10]. The second was performed in South Africa and reported in 2015, on a man who lost his penis in a failed ritual circumcision; results have been described as satisfactory, with the recipient reporting natural spontaneous erections and impregnating his partner [11, 12]. All three patients were able to void spontaneously through the urethra.

Following the first case of penile transplantation, authors in [8, 13] criticized the approach adopted in patient selection and possibly some aspects of the surgical technique used by the Chinese group. Nevertheless, penile transplantation was still believed to potentially offer the best outcomes in penile reconstruction [8].

With this vision in mind, recent research is investigating the anatomical structures and the feasibility of penile transplantation; so far, previous research has not focused specifically on penile explantation with the purpose of penile construction for trans-men.

This study aims to determine whether an en bloc surgical technique can be employed for penile transplantation from a cadaveric donor male to a recipient with female genitalia for trans-men GAS. Options for explantation of the penis and associated vessels, nerves, and urethra are investigated.

## 2. Materials and Methods

### 2.1. Subjects

Subjects of the study are three male cadavers preserved in a phenol-ethanol solution. They have been provided by the Department of Medical Biochemistry and Cell Biology at Sahlgrenska Academy, University of Gothenburg. Their ages were not provided, but all subjects were elderly at their deaths. The cadavers had been dissected for teaching purposes prior to the dissections in this study but had only been moderately dissected in the genital area. More specifically, the groin area was partially dissected to expose vessels; the abdomens had been opened to show internal organs; and the scrotums had been opened on one side and one spermatic cord was removed.

### 2.2. Ethics for the Cadaver Dissection

It is not within the purpose of this manuscript to discuss the ethics of penis transplantation. Ethical issues related to penis transplantation have been initially announced by Caplan et al. [14]

The subjects of this study are cadavers willingly donated to Karolinska Institutet for teaching and research purposes. No reservations or caveats regarding genital dissection or transsexual research were made by the donors.

The Department of Medical Biochemistry and Cell Biology at the Sahlgrenska Academy, University of Gothenburg, operates under a statutory right to conduct anatomical research on donated bodies. This is replacing and waiving a IRB approval specific for the present study.

The identity of the donors is protected and no identifying information whatsoever has been available to the researchers.

### 2.3. Method

The study was conducted in the form of explorative dissections on the genital and pelvic regions of the cadavers, which were placed in a dorsal recumbent position.

## 3. Results

### 3.1. Dissection of the First Specimen

On the first specimen, the dissection was begun by identifying the internal iliac vessels in the abdomen. Dissection of the perineal area was carried out in a manner similar to the approach used to reach the prostate during trans-perineal prostatectomy or to create a cavity during vaginoplasty for trans-women: the surgical dissection went through the perineum, above the rectum, and toward the prostate; then perineal structures as pudendal nerves and internal pudendal vessels were located.

While dissecting the groin areas, one of the external pudendal vessels on the left hand side could be identified and dissected with a patch from the femoral artery. Remaining external pudendal vessels could not be identified due to previous dissection of the cadaver for educational purposes.

The testicles and spermatic cords were removed from the scrotum. The next step was to dissect downwards from the abdomen to reach the groin area, in the plane between the urinary bladder and prostate ventrally and the colon and rectum dorsally, in order to be able to pull the pelvic structures (vessels, urinary bladder, and prostate, with the ureters transected) out through the perineum. Branches of the internal iliac arteries that appeared to be going to nongenital structures were transected.

To free the transplant, the dissection was continued above the penis down to the pubic symphysis. The suspensory ligament of the penis was transected, and the dissection continued along the pubic arch to free the penile bodies and the entire transplant.

The transplant unit was then removed and placed on the back table; next step was to separate the prostate and urinary bladder from the specimen without causing damage to the urethra, nerves, or vessels. At this point, it became visible that the dissection had only spared one iliac branch on each side, and as these spread diffusely in the bladder-prostate complex, it appeared more likely that they were the inferior vesical arteries than the internal pudendal arteries that were intended to be retrieved.

**Figure 1:**
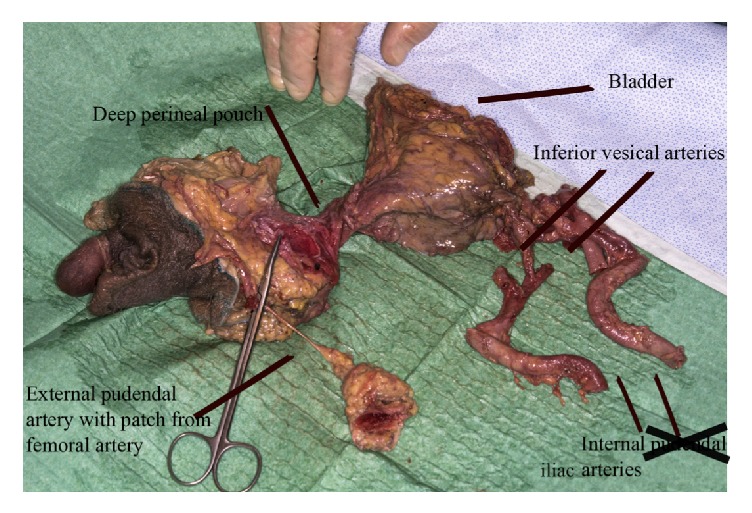
Explant from first specimen. The section containing the penis and the section containing the bladder and prostate are connected by a cordlike structure described as the deep perineal pouch. This is containing muscular structures, branches of the internal pudendal artery and vein, branches of the perineal nerves, and the membranous urethra. It is not possible to identify the internal pudendal vessels.

The section of the explant containing the penis and the section containing the bladder and prostate are connected by a cordlike structure described as the deep perineal pouch containing muscular structures, branches of the internal pudendal artery and vein, branches of the perineal nerves, and the membranous urethra. This section was dissected in an attempt to locate structures in order to free the prostate and bladder from the specimen. The urethra could be identified but not the internal pudendal vessels (Figure 1).

Thus, the first dissection failed in explanting the necessary structures intact from the male specimen.

### 3.2. Dissection of the Second Specimen

The second male specimen was dissected more proximally, without removal of the pelvic structures. It was attempted to explant the genitals and to identify the relevant vessels and nerves without abdominal dissection. The dissection was carried out down to the suspensory ligament of the penis, similarly to the first specimen. Differently from the first dissection, in the second specimen the corpora were transected. The inguinal dissection was then conducted to free the penis from the perineum, as in the first dissection. However, upon back table dissection, it was visually determined that relevant vessels and the urethra had dimensions that were considered inadequate for transplantation onto female genitalia.

### 3.3. Dissection of the Third Specimen

For the dissection of the third male specimen, another approach for explantation was explored. First, external pudendal vessels were attempted to be identified, dissected, and followed to where they spread in the skin. On the right hand side, one external pudendal artery could be identified and dissected (superficial external pudendal artery); another transected vessel was identified below, likely to be the deep external pudendal artery. One external pudendal vein could also be identified, though it had been transected in the previous dissections.

On the left hand side, one external pudendal artery could be identified, passing below the femoral vein and into the skin.

The vessels were dissected and removed with a patch from the femoral artery.

Then, the spermatic cord on the left hand side was identified and cut above the point where the vessels crossed; the testicle was left in place, since it was determined it could just as well be removed at a later stage.

Next, the perineum was dissected. The crura of the corpora cavernosa were located. The bundle of vessels associated with the crura was assumed to contain the internal pudendal vessels.

**Figure 2:**
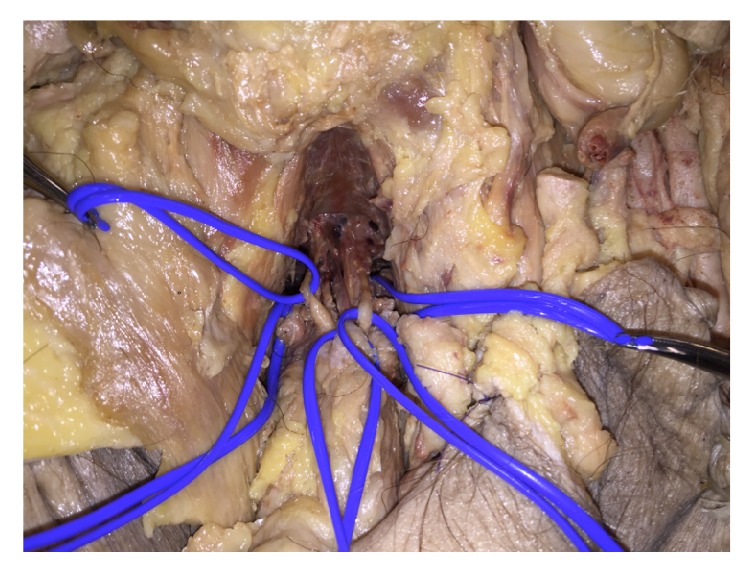
Identification of deep dorsal vein and dorsal nerves in the third specimen. Clockwise from the right in vessel loops: left crura of corpus cavernosum, left dorsal nerve, deep dorsal vein, right internal pudendal vessels, and right dorsal nerve.

The skin was incised similarly to the dissection of the first male. Dissection was continued above the penis, down to the pubic symphysis, with transection of the suspensory ligament, location of the dorsal penile vein, and dorsal nerves and continued dissection along the pubic arch (Figure 2).

The bulb of the corpus spongiosum was identified through locating the bladder and prostate in the abdomen. The deep perineal pouch could be identified between the bulb and the prostate and transected. Vessels, nerves, and soft tissue were transected. The explant was removed and dissected separately on the back table.

**Figure 3:**
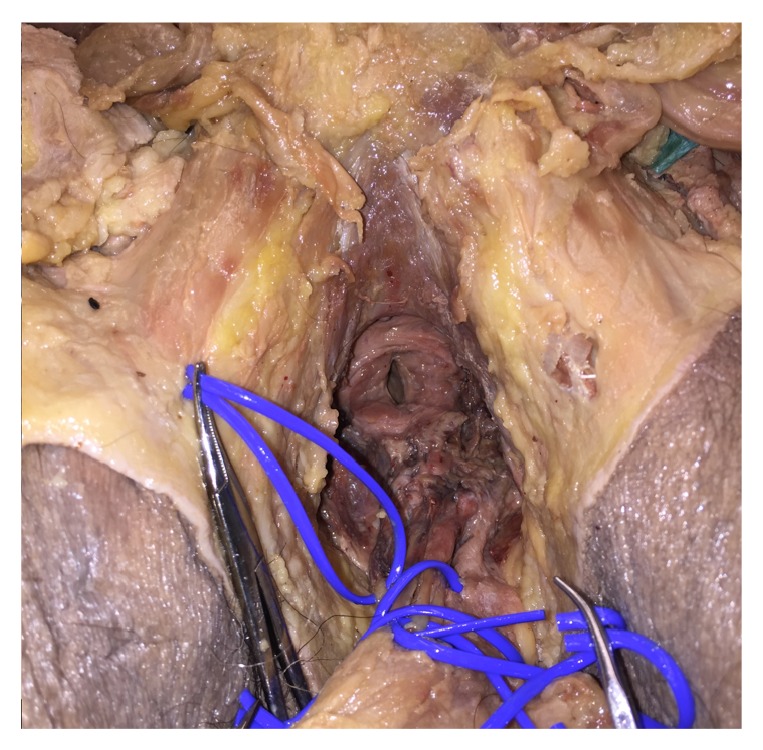
Transection of urethra, nerves, and vessels during explantation of genitals from the third specimen. Lumen of urethra entering prostate visible.

**Figure 4:**
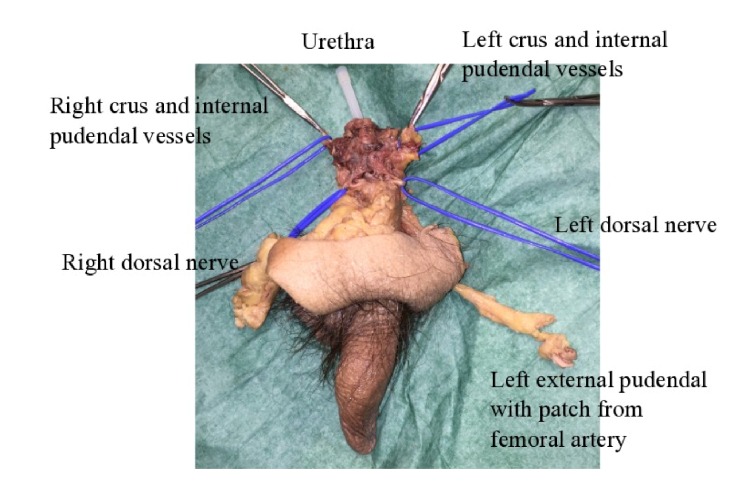
Explant from third specimen, showing penis; right and left dorsal nerves; right and left crus and internal pudendal vessels; left external pudendal artery with a patch from the femoral artery; and urethra (with needle cover inserted).

The dissection of the third male specimen thus succeeded in explanting the relevant structures en bloc, with a note that not all the external pudendal vessels could be identified due to the state of the cadaver (Figures 3 and 4).

## 4. Discussion

Through the dissections of the three male specimens, a method was developed to explant the male genitalia and associated structures en bloc.

The dissection of the first specimen was unsuccessful in preserving necessary vessels, due to difficulties in locating the internal pudendal vessels. The dissection of the second specimen failed in preserving adequate lengths of the necessary structures. Finally, in the third specimen, male genitalia were explanted by dissection along the pubic arch and through the perineum to locate the crura of the corpora cavernosa, which were freed from the bone, and the internal pudendal vessels, which were transected. The suspensory ligament of the penis, the dorsal penile nerves, and the deep dorsal vein were identified and transected just below the symphysis. The prostate and bulb of the corpus spongiosum were identified between and superior to the crura, and the deep perineal pouch containing the membranous urethra was transected. The external pudendal vessel which could be identified was followed from the femoral artery to where they branched in the inguinal skin, which was included in the transplant.

In the third specimen, the internal pudendal vessels were transected close to the crura of the corpora cavernosa. It was suggested that instead the abdomen could be dissected similarly to in the dissection of the first male specimen, and a greater length of the vessels was preserved, by following the branches of the internal iliac vessels and meeting the dissection of the perineum. This would ensure preservation of a length of vessel adequate to reach the recipient's vessels for anastomosis. Further, if the vessels are transected close to the crura, the posterior scrotal arteries (which branch further dorsally in the pelvis) will not be included in the explant, and vascularization to the skin of the explant is not entirely guaranteed. This means that abdominal dissection might be necessary to avoid necrosis of the skin.

It is possible that there are additional options for surgical technique of explantation which have yet to be attempted and which could come to light with future dissections. For example, it was suggested that the vasculature might be more accessible if the most ventral part of the pubic arch was sawed off. In addition, the question of whether the erectile tissue of the transplant and recipient could be aligned has not been investigated.

It should be noted that vascular anatomy is subject to individual variation. The internal pudendal vessels, for example, can have an aberrant course or have accessory vessels. In some cases, an accessory vessel may be solely responsible of the blood supply to the corpora cavernosa [15, 16]. Radiographic imaging of the vasculature of prospective recipients will likely be necessary.

### 4.1. Previous Anatomical Studies

In 2014, Tuffaha et al. conducted a cadaveric study of the perfusion territories of the arteries of the penis to find the cause behind skin necrosis following penile replantation and to find surgical options for penile transplantation to natal males. They concluded that the primary reason for skin necrosis after replantation is that the external pudendal arteries, which branch in the groin area and generally cannot be repaired after traumatic penile amputation, are responsible for supplying blood to the greater part of the penile shaft skin. It was suggested that the external pudendal system, which is easily identified, should be included in the case of proximal penile transplantation [15, 17].

In 2016, Tiftikcioglu et al. presented a cadaveric dissection study to investigate the anatomic feasibility of penile transplantation: seventeen male cadavers were dissected to reveal detailed anatomy of the dorsal neurovascular structures including dorsal arteries, superficial and deep dorsal veins, and dorsal nerves of the penis. They concluded that the level of harvest should be determined according to the extension of the defect, where a cis-male patient with a proximal penile defect will receive a partial shaft allograft, while a transgender patient will receive a total allograft [18]. More specifically for the latter, the penis must be harvested deep to its root at the hilum where the bulbar and ischiocavernosal muscles sit. The arterial dissection should continue retrograde until internal pudendal artery is reached so all the branches, dorsal, cavernosal, and bulbourethral arteries, can be included in the allograft. Internal pudendal artery should be divided at a point after it has given its rectal branches [18]. Dorsal nerve dissection should start at the penile root and proceed to perineum with care, on the same plane with the internal pudendal artery and vein. Nerve harvest does not need to proceed too far, as it will be coapted to the dorsal clitoral nerve [18]. In the trans-man recipient, female urethral length can be performed for anastomosis [18], as it is already commonly performed in association with other techniques for penis reconstruction as, for example, radial forearm flap [19].

### 4.2. Knowledge from the Current Techniques for Penile Reconstruction in GAS

The possible gold standard for phalloplasty for trans-men with gender dysphoria might be represented by free radial forearm flap [3]. With this technique, perfusion is ensured by microsurgical anastomosis of the radial artery end-to-side to the femoral artery and the cephalic vein to the saphena magna. Neural sensation is accomplished by connecting forearm cutaneous nerves to one dorsal nerve of the clitoris, leaving the other intact, and to one ilioinguinal nerve, allowing the neophallus to have both tactile and erogenous sensation. The clitoris is not removed, but deepithelialized, freed from its ligaments, and repositioned at the base of the penis, ensuring erogenous sensation and capability to achieve orgasm [3, 4, 20, 21].

In metoidioplasty, the clitoris is similarly freed from the clitoral ligaments, and the urethral plate was divided, in order to lengthen and straighten the constructed phallus. The clitoris retains its erogenous sensitivity in this manner [22].

In both phalloplasty for trans-men and metoidioplasty, the pars fixa of the neourethra is constructed by using the labia minora and eventually a buccal mucosa graft, while other flaps (e.g., tubularized radial forearm flap) are used for the reconstruction of the pars pendulans [4, 6]. Urinary complications (e.g., fistula, stenosis) following urethra reconstruction are high [3, 4, 6, 23].

### 4.3. Remaining Questions

There are a number of issues that need to be addressed before penile transplantation could become an option for GAS. First, it should be investigated to which extent the trans-male population is interested in penile transplantation and whether a potential recipient could psychologically accept a transplanted penis as their own. A pilot study with an initial assessment of this issue is confirming some interest from the trans-men recipient population [2]. Second, ethical issues are as follows: is the benefit (improved quality of life) versus risk (of life-time immunosuppression) ratio favorable? Is there an ethical issue in retrieving genital organs from donors who may have not consented specifically to this type of donation? How to justly allocate public resources, in both research and clinical care?

Additional necessary steps to consider are animal research, radiographic imaging of vascular anatomy and mapping of variant anatomies, and live explantation trials. Likely, further cadaveric research will be required as well. For example, it needs to be established which vessels are appropriate for anastomosis in the recipient, the femoral and epigastric vessels being the candidates closest at hand.

### 4.4. Methodological Considerations

Inductive reasoning from the available knowledge on anatomy and urogenital and transsexual surgery has been employed to construct a theory on how the transplant could achieve perfusion and sensation, but it will remain a theory until tested in a live setting. In addition, the very small number of specimens makes it impossible to draw empirical conclusions regarding the feasibility of the method.

The present study demonstrated the possibility to explant the penis and associated vessels, nerves, and urethra en bloc from a cadaver. We thus suggest a surgical technique for en bloc explantation aiming for transplantation of the penis from a cadaveric donor male to a recipient with female genitalia.

This, being a starting point for research into penile transplantation in trans-men GAS, will obviously need further research before becoming a clinical reality.
